# Association between perceived school climate and employment anxiety among medical undergraduates: the mediating role of intentional self-regulation and the moderating role of perceived employability

**DOI:** 10.3389/fpsyt.2026.1927113

**Published:** 2026-09-15

**Authors:** Nana Sui, Cheng Zhang, Yanjuan Zhou, Shuyu Liu, Botang Guo, Yuchun Wang, Yuan Cao, Peipei Cai, Junhui Yan, Zhanpeng Qi

**Affiliations:** 1 Pathology School, Qiqihar Medical University, Qiqihar, China; 2 School of Public Health, Harbin Medical University, Harbin, China; 3 School of Marxism, Harbin Medical University, Harbin, China; 4 Center for Affective Disorders, Harbin First Specialized Hospital, Harbin, Heilongjiang, China; 5 Department of General Practice, The Affiliated Luohu Hospital of Shenzhen University Medical School, Guangdong Province, Shenzhen, China; 6 School of Pharmacy, Qiqihar Medical University, Qiqihar, China; 7 Department of Accounting and Finance, Heilongjiang Polytechnic, Harbin, Heilongjiang, China; 8 Organization Department, Qiqihar Medical University, Qiqihar, China

**Keywords:** employment anxiety, intentional self-regulation, moderated mediation, perceived employability, perceived school climate

## Abstract

**Background:**

Employment anxiety is an important psychological concern for medical undergraduates during the transition from university to work. This study examined the association between perceived school climate and employment anxiety and evaluated the roles of intentional self-regulation and perceived employability in this association.

**Methods:**

A cross-sectional survey was conducted among 3,857 undergraduate students from four medical universities in Heilongjiang Province, China, between September and December 2025. Participants completed measures of perceived school climate, intentional self-regulation, perceived employability, employment anxiety, and demographic and academic characteristics. Hierarchical regression, indirect-association analysis, and moderated mediation analysis with 5,000 bootstrap resamples were performed. Multivariable models included university fixed effects and were adjusted for demographic and academic covariates.

**Results:**

A more positive perceived school climate was associated with lower employment anxiety and stronger intentional self-regulation. Intentional self-regulation statistically accounted for part of the association between perceived school climate and employment anxiety. Perceived employability moderated the association between intentional self-regulation and employment anxiety: the negative association, and consequently the conditional indirect association, was stronger among students with lower perceived employability and weaker among those with higher perceived employability. However, the incremental magnitude of the moderation effect was modest.

**Conclusions:**

Supportive perceived school climate was associated with lower employment anxiety partly through intentional self-regulation, with a modest moderating role of perceived employability. Integrating school-climate improvement, self-regulation training, and targeted career support may strengthen psychological adaptation during the school-to-work transition.

## 1 Introduction

Employment anxiety during the transition from university to work has become an important psychological concern among university students. Late adolescence and emerging adulthood involve simultaneous demands related to identity development, increasing autonomy, and preparation for occupational roles. During this period, students must make consequential decisions under conditions of academic pressure, economic uncertainty, and changing labor-market expectations. Recent evidence indicates that young people’s mental health is influenced not only by individual vulnerability but also by educational pressure, economic insecurity, social uncertainty, and access to psychological support (1). Anxiety during this developmental stage should therefore be understood as both a mental health concern and a problem of developmental adaptation (2). Employment anxiety refers to anxiety-related responses arising from anticipated job uncertainty, perceived competition, concerns about occupational competence, and doubts about future career adaptation. Studies among Chinese university students have linked employment-related stress with broader psychological distress (3), while recent scale-development studies support employment anxiety as a distinguishable and measurable construct in higher education research (4, 5). Recent evidence further suggests that academic stress may be associated with employment anxiety partly through individual psychological resources such as resilience (6). However, employment anxiety is unlikely to arise solely from objective labor-market conditions. It may also reflect how students perceive their educational environment, regulate career-related goals, and evaluate their own ability to obtain and maintain employment.

Medical undergraduates represent a particularly relevant population in which to examine these processes. Medical education involves prolonged professional preparation, intensive academic demands, frequent examinations, and complex postgraduation decisions. Students may need to choose among postgraduate education, standardized residency training, clinical employment, and further specialization, often while facing uncertainty regarding geographic mobility, specialty selection, and future professional development. Reviews consistently indicate a substantial burden of anxiety, depression, stress, and psychological distress among medical students (7). Educational demands and the quality of the learning environment have also been identified as important correlates of their mental health (8). Employment anxiety among medical undergraduates should therefore be examined not only as a response to labor-market competition but also as an outcome arising from the interaction between educational context and individual developmental resources.

Relational developmental systems theory provides an overarching framework for understanding this interaction. The theory proposes that adaptive development emerges from reciprocal relations between individuals and their contexts rather than from either personal characteristics or environmental conditions alone (9). Positive youth development research emphasizes that adaptive development arises from the alignment between contextual developmental assets and individual strengths (10). Supportive educational settings may provide interpersonal support, fairness, belonging, and developmental opportunities, whereas individual capacities influence how students use these resources when responding to developmental challenges. From this perspective, employment anxiety may be understood as an adaptation outcome shaped by both perceived school climate and students’ capacity to regulate career-related goals.

Perceived school climate represents students’ appraisal of the social and organizational qualities of their educational environment, including support, fairness, connectedness, and interpersonal relationships. A positive school climate may be associated with lower employment anxiety through several pathways. Supportive relationships may provide emotional security during periods of uncertainty, institutional resources may improve students’ access to information and guidance, and a stronger sense of belonging may enhance confidence in managing future transitions. Longitudinal research has linked positive school climate with fewer emotional and behavioral difficulties and better mental wellbeing (11), and school-climate improvement has been proposed as a population-level approach to youth mental health promotion (12, 13). Nevertheless, school climate is a relatively distal contextual resource. Its association with employment anxiety is likely to depend on whether students are able to translate educational support into goal-directed responses to career uncertainty.

Intentional self-regulation may provide this linking mechanism. It refers to the capacity to select personally meaningful goals, optimize available resources, adjust behavioral strategies, and compensate for barriers when developmental circumstances change. Within positive youth development theory, intentional self-regulation is regarded as an individual strength through which young people actively coordinate their needs and goals with opportunities available in their environment (14). This concept provides a theoretical bridge between school climate and employment anxiety. A supportive school environment may facilitate intentional self-regulation by providing guidance, feedback, social support, and opportunities for goal exploration. Students may then use these resources to clarify occupational goals, identify available pathways, revise unrealistic plans, and develop alternative strategies when facing career-related obstacles.

Intentional self-regulation may, in turn, be associated with lower employment anxiety because it increases students’ perceived control over uncertain career tasks. Medical undergraduates with stronger self-regulatory capacity may be better able to decompose complex career decisions into manageable goals, mobilize available educational resources, and modify their plans when preferred options are unavailable. Evidence has linked self-regulation with learning engagement among young people (15). These findings suggest that perceived school climate may be associated with employment anxiety partly because supportive educational contexts facilitate the self-regulatory capacities needed to manage the school-to-work transition. Intentional self-regulation is therefore positioned as a mediator because it represents the individual process through which a distal educational context may be translated into career-related psychological adaptation.

However, the association between intentional self-regulation and employment anxiety may vary across students. From a positive youth development perspective, adaptive outcomes arise from the interplay between individual strengths and career-related resources. Perceived employability represents a career-specific appraisal of students’ employment competence and future job prospects and has been associated with academic commitment, competency, and well-being (16). Its association with self-motivated learning strategies further suggests that employability appraisals may influence how students mobilize their self-regulatory resources (17). Moreover, perceived employability may change across time and employment contexts, supporting its interpretation as a context-sensitive career resource (18). Perceived future employability has also been associated with lower future anxiety among healthcare management students (19). When perceived employability is low, students may rely more heavily on intentional self-regulation to organize career goals, manage uncertainty, and respond to perceived employment barriers. In contrast, higher perceived employability may provide greater confidence and perceived control, reducing the relative contribution of self-regulation. Accordingly, the negative association between intentional self-regulation and employment anxiety was expected to be stronger at lower levels of perceived employability and weaker at higher levels.

Based on relational developmental systems theory and the distinction between contextual, self-regulatory, and career-specific resources, the present study tested a conditional process model linking perceived school climate with employment anxiety among medical undergraduates. The following hypotheses were proposed:

H1: A more positive perceived school climate is associated with lower employment anxiety among medical undergraduates.

H2: Perceived school climate has an indirect association with employment anxiety through intentional self-regulation, such that a more positive school climate is associated with stronger intentional self-regulation, which is in turn associated with lower employment anxiety.

H3: Perceived employability moderates the association between intentional self-regulation and employment anxiety. The negative association is expected to be stronger at lower levels of perceived employability and weaker at higher levels of perceived employability.

H4: The indirect association between perceived school climate and employment anxiety through intentional self-regulation is conditional on perceived employability. The indirect association is expected to be stronger at lower levels of perceived employability and weaker at higher levels of perceived employability.

By integrating educational context, intentional self-regulation, and career-specific appraisal within one conditional process model, this study seeks to clarify not merely whether these variables are associated, but how and under what conditions perceived school climate is related to employment anxiety. The findings may provide a more theoretically differentiated basis for future longitudinal research and for the development of coordinated educational, self-regulatory, and career-support strategies during the transition from medical education to work.

## 2 Methods

### 2.1 Study design and participants

This cross-sectional study was conducted from September to December 2025 in four universities offering undergraduate medical programs in Heilongjiang Province, China, including Harbin Medical University, Qiqihar Medical University, Mudanjiang Medical University, and Jiamusi University. Undergraduate medical students from first to fifth year were selected as the target population. A stratified cluster sampling strategy was adopted. The four participating universities were used as sampling strata, and approximately 1,000 questionnaires were distributed in each university. Within each university, students from different academic years were invited to participate to ensure coverage of the full undergraduate training stage. Data were collected using an anonymous online questionnaire distributed through university-based student management or class communication channels. Participants completed the questionnaire independently by scanning the questionnaire link or QR code.

The inclusion criteria were as follows: full-time undergraduate medical students enrolled in one of the participating universities, ability to understand and independently complete the questionnaire, and voluntary agreement to participate in the survey. Students were excluded if they submitted questionnaires with missing key variables, clearly inconsistent responses, duplicate responses, extremely short completion time, or obvious regular response patterns suggesting invalid completion. A total of 4,000 questionnaires were distributed. After excluding invalid questionnaires, 3,857 valid responses were included in the final analysis, yielding an effective response rate of 96.43%. The study was conducted in accordance with the principles of voluntary participation, anonymity, and confidentiality. Before completing the questionnaire, participants were informed of the study purpose, data confidentiality, voluntary nature of participation, and their right to withdraw at any time. All participants were aged 18 years or older and provided electronic informed consent before completing the questionnaire. Ethical approval was obtained from the Ethics Committee of Qiqihar Medical University (Approval No. 20253).

### 2.2 Measures

#### 2.2.1 Demographic and academic characteristics

Demographic and academic characteristics were collected using a structured questionnaire. The variables included age, sex, grade, residence, only-child status, family economic status, academic pressure, internship or clinical exposure experience, and intention to take the postgraduate entrance examination. Age was recorded as a continuous variable. Sex was categorized as male or female. Grade was categorized from first year to fifth year. Residence was categorized as rural or urban. Only-child status, internship or clinical exposure experience, and postgraduate entrance examination intention were recorded as binary variables. Family economic status and academic pressure were assessed using ordered response categories.

#### 2.2.2 Perceived school climate

Perceived school climate was assessed using Part I of the Delaware School Survey-Student, namely the School Climate Scale. The scale was developed and validated in previous work on the Delaware School Climate Survey (20). The scale contains 30 scored items assessing students’ perceptions of school climate, including student engagement, teacher-student relationships, student-student relationships, fairness of school rules, school safety, bullying prevention, social-emotional support, and the overall school environment. One attention-check item was included in the original questionnaire but was not included in the total score. Each item was rated on a 4-point Likert scale: 1 = strongly disagree, 2 = disagree, 3 = agree, and 4 = strongly agree. Negatively worded items were reverse scored before calculating the total score. Specifically, items B9, B14, and B26 were reverse-coded. The total score was calculated by summing the 30 scored items, with possible scores ranging from 30 to 120. Higher scores indicated a more positive perceived school climate. In the present study, Cronbach’s alpha was 0.941, and confirmatory factor analysis showed good model fit (CFI = 0.994, TLI = 0.993, RMSEA = 0.014, and SRMR = 0.012).

#### 2.2.3 Intentional self-regulation

Intentional self-regulation was measured using the Likert-type 18-item version of the Selection, Optimization, and Compensation Questionnaire reported by Geldhof et al. (21). The scale assesses three core components of intentional self-regulation: selection, optimization, and compensation. Selection refers to identifying and prioritizing important goals; optimization reflects the use of strategies, resources, and effort to pursue goals; and compensation refers to adjusting strategies or finding alternative pathways when facing difficulties or constraints. Each item was rated on a 5-point Likert scale: 1 = strongly disagree, 2 = disagree, 3 = neutral, 4 = agree, and 5 = strongly agree. The total score was calculated by summing all 18 items, with possible scores ranging from 18 to 90. Higher scores indicated stronger intentional self-regulation. In the present study, Cronbach’s alpha was 0.930, and confirmatory factor analysis showed good model fit (CFI = 0.997, TLI = 0.997, RMSEA = 0.014, and SRMR = 0.010).

#### 2.2.4 Perceived employability

Perceived employability was assessed using the Student Self-perceived Employability Scale developed by Rothwell et al. (22). The scale contains 16 items and measures university students’ perceptions of their employability. It covers students’ perceived academic performance, engagement with study and career preparation, confidence in obtaining employment, perceived competitiveness in the labor market, and perceived external value of their university and field of study. Each item was rated on a 5-point Likert scale: 1 = strongly disagree, 2 = disagree, 3 = neutral, 4 = agree, and 5 = strongly agree. The total score was calculated by summing all 16 items, with possible scores ranging from 16 to 80. Higher scores indicated greater perceived employability. In the present study, Cronbach’s alpha was 0.925, and confirmatory factor analysis showed good model fit (CFI = 0.998, TLI = 0.998, RMSEA = 0.012, and SRMR = 0.010).

#### 2.2.5 Employment anxiety

Employment anxiety was measured using the Employment Anxiety Scale for Chinese College Graduates developed by Wang and Hu (5). The final scale contains 14 items and includes two dimensions: cognitive expectations and somatic symptoms. The scale assesses anxiety related to future employment, including worry about career prospects, concern about insufficient job-seeking skills, uncertainty regarding career choice, perceived pressure from employment competition, and anxiety-related physical or emotional responses during the school-to-work transition. Each item was rated on a 5-point Likert scale: 1 = strongly disagree, 2 = disagree, 3 = neutral, 4 = agree, and 5 = strongly agree. The total score was calculated by summing all 14 items, with possible scores ranging from 14 to 70. Higher scores indicated higher levels of employment anxiety. In the present study, Cronbach’s alpha was 0.920, and confirmatory factor analysis showed good model fit (CFI = 0.999, TLI = 0.999, RMSEA = 0.010, and SRMR = 0.009). Detailed results regarding factor structure, reliability, convergent validity, discriminant validity, and item-level performance of all above scales are presented in **Supplementary Tables 1**-**5**.

### 2.3 Quality control

Several procedures were used to ensure data quality. The questionnaire consisted of full versions of established instruments and a demographic and academic information module. Before formal data collection, the questionnaire was reviewed by the research team for item clarity, logical consistency, and completion burden in the context of medical undergraduates. During data collection, all questionnaires were completed anonymously and independently by participants. Uniform instructions were provided at the beginning of the questionnaire to reduce misunderstanding and response bias. Required-response settings were used for key variables to reduce missing data, and duplicate submissions were restricted where technically feasible. After data collection, questionnaires with missing key variables, duplicate submissions, extremely short completion time, failed attention checks, logical contradictions, or highly repetitive response patterns were excluded before analysis.

### 2.4 Statistical analysis

Statistical analyses were performed using IBM SPSS Statistics version 26.0, PROCESS macro version 4.0, and R version 4.2.3. Continuous variables are presented as means and standard deviations, whereas categorical variables are presented as frequencies and percentages. Differences in employment anxiety across demographic and academic characteristics were examined using independent-samples *t* tests for variables with two categories and one-way analysis of variance for variables with three or more categories. When an omnibus analysis of variance was statistically significant, Tukey-adjusted *post-hoc* comparisons were conducted, with mean differences, adjusted *P* values, and 95% confidence intervals reported. Pearson correlation coefficients were calculated to examine bivariate associations among the main study variables. Common method bias was assessed using Harman’s single-factor test and confirmatory factor analysis, in which the theoretically specified four-factor model was compared with a one-factor model. Confirmatory factor analyses were conducted to examine the measurement structure of the four scales, and model fit was evaluated using CFI, TLI, RMSEA, and SRMR.

Multiple linear regression models were used to examine the associations among perceived school climate, intentional self-regulation, perceived employability, and employment anxiety. All multivariable models were adjusted for age, sex, grade, residence, only-child status, family economic status, academic pressure, internship experience, and postgraduate entrance examination intention. Multicategory covariates were modelled as categorical variables, and university fixed effects were included in all multivariable models. Leave-one-university-out sensitivity analyses were conducted to assess whether the principal findings were driven by any single institution. The mediating role was examined using PROCESS Model 4. The indirect effect was estimated using 5,000 bootstrap resamples. Both unstandardized and completely standardized indirect effects were reported with their corresponding 95% bootstrap confidence intervals. An indirect association was considered statistically supported when the confidence interval did not include zero. The moderated mediation model was examined using PROCESS Model 14. Intentional self-regulation and perceived employability were mean-centered before constructing the interaction term. Conditional effects and conditional indirect effects were estimated at one standard deviation below the mean, the mean, and one standard deviation above the mean of perceived employability. The index of moderated mediation was estimated using 5,000 bootstrap resamples and was considered statistically supported when its 95% bootstrap confidence interval excluded zero. HC3 heteroskedasticity-robust standard errors and 95% confidence intervals were used for coefficient-level inference in the final regression models. Effect magnitude was evaluated using standardized and unstandardized regression coefficients, R², adjusted R², and the change in explained variance after inclusion of the interaction term. Model assumptions were assessed using variance inflation factors, the Breusch-Pagan test, the Ramsey RESET test, and residual skewness and kurtosis. Subgroup differences were evaluated using interaction terms. All statistical tests were two-sided, and *P* < 0.05 was considered statistically significant.

## 3 Results

### 3.1 Participant characteristics and employment anxiety

A total of 3,857 undergraduate medical students were included in the final analysis. The mean age was 20.06 years (SD = 1.51), 2,343 participants (60.7%) were female, and the mean employment anxiety score was 41.51 ± 9.95. The demographic and academic characteristics of the participants and the corresponding employment anxiety scores are presented in **Table 1**. Employment anxiety differed significantly across grade, family economic status, academic pressure, and university affiliation in the omnibus analyses (all *P* < 0.05). No statistically significant differences were observed according to age group, sex, residence, only-child status, internship experience, or postgraduate entrance examination intention. Tukey-adjusted *post-hoc* analyses showed that none of the pairwise grade comparisons remained statistically significant. Students with low family economic status reported higher employment anxiety than those with middle or high family economic status. Students reporting high academic pressure had higher employment anxiety than those reporting low or moderate pressure. University D had a higher mean employment anxiety score than University C, whereas the remaining pairwise university comparisons were not statistically significant. Detailed *post-hoc* results are provided in **Supplementary Table 6**. University-specific analyses showed no significant differences in perceived school climate, intentional self-regulation, or perceived employability across the four institutions. Employment anxiety differed across universities (*F* = 3.273, *P* = 0.020), consistent with the *post-hoc* results described above (**Supplementary Table 7**).

**Table 1:** Participants’ characteristics and the distribution of employment anxiety.

Variables	N (%)	Employment anxiety, Mean ± SD	*F/t*	*P*
Age group			2.037	0.131
≤19	1413 (36.6)	41.79 ± 9.78		
20-21	1737 (45.0)	41.15 ± 10.15		
≥22	707 (18.3)	41.82 ± 9.79		
Sex			0.664	0.506
Male	1514 (39.3)	41.64 ± 9.94		
Female	2343 (60.7)	41.42 ± 9.96		
Grade			2.696	0.029
First-year	558 (14.5)	41.88 ± 9.82		
Second-year	761 (19.7)	42.29 ± 9.77		
Third-year	986 (25.6)	41.03 ± 9.95		
Fourth-year	996 (25.8)	41.02 ± 10.21		
Fifth-year	556 (14.4)	41.80 ± 9.83		
Residence			-1.308	0.191
Urban	1867 (48.4)	41.29 ± 9.84		
Rural	1990 (51.6)	41.71 ± 10.06		
Only child			1.778	0.075
Yes	1500 (38.9)	41.87 ± 10.10		
No	2357 (61.1)	41.28 ± 9.86		
Family economic status			5.511	0.004
Low	1018 (26.4)	42.35 ± 9.99		
Middle	1796 (46.6)	41.35 ± 9.82		
High	1043 (27.0)	40.96 ± 10.11		
Academic pressure			23.252	<0.001
Low	912 (23.6)	40.12 ± 9.89		
Moderate	1481 (38.4)	41.08 ± 9.79		
High	1464 (38.0)	42.82 ± 10.01		
Internship experience			1.370	0.171
No	2735 (70.9)	41.65 ± 9.89		
Yes	1122 (29.1)	41.17 ± 10.11		
Postgraduate entrance examination intention			1.263	0.207
No	1812 (47.0)	41.73 ± 9.90		
Yes	2045 (53.0)	41.32 ± 10.00		
University affiliation			3.273	0.020
University A	921 (23.9)	41.10 ± 10.30		
University B	1034 (26.8)	41.66 ± 9.63		
University C	961 (24.9)	41.00 ± 9.96		
University D	941 (24.4)	42.27 ± 9.92		

*t* tests were used for two-group comparisons and one-way ANOVA was used for variables with three or more groups. *P* < 0.05 was considered statistically significant. Detailed Tukey-adjusted *post-hoc* comparisons are presented in **Supplementary Table 6**.

### 3.2 Descriptive statistics, reliability, and correlations

**Table 2** summarizes the descriptive statistics, internal consistency, and correlations among the study variables. The mean scores of perceived school climate, intentional self-regulation, perceived employability, and employment anxiety were 89.95 ± 12.88, 59.80 ± 18.51, 51.81 ± 10.57, and 41.51 ± 9.95, respectively. The Cronbach’s alpha coefficients for perceived school climate, intentional self-regulation, perceived employability, and employment anxiety were 0.941, 0.930, 0.925, and 0.920, indicating good internal consistency. Pearson correlation analysis showed that perceived school climate was positively correlated with intentional self-regulation (*r* = 0.260, *P* < 0.01) and perceived employability (*r* = 0.339, *P* < 0.01), and negatively correlated with employment anxiety (*r* = -0.277, *P* < 0.01). Intentional self-regulation was positively correlated with perceived employability (*r* = 0.073, *P* < 0.01) and negatively correlated with employment anxiety (*r* = -0.250, *P* < 0.01). Perceived employability was also negatively correlated with employment anxiety (*r* = -0.211, *P* < 0.01).

**Table 2:** Descriptive statistics and correlation analysis of each variable.

No.	Variable	*M*	*SD*	*α*	1	2	3	4	5
1	Age	20.06	1.51	-	1				
2	Perceived school climate	89.95	12.88	0.941	0.012	1			
3	Intentional self-regulation	59.80	18.51	0.930	0.012	0.260**	1		
4	Perceived employability	51.81	10.57	0.925	0.025	0.339**	0.073**	1	
5	Employment anxiety	41.51	9.95	0.920	-0.008	-0.277**	-0.250**	-0.211**	1

Correlations are Pearson correlation coefficients. *P < 0.05, **P < 0.01.

### 3.3 Common method bias assessment

**Supplementary Figure 1** and **Supplementary Table 8** summarize the assessment of common method bias. Harman’s single-factor test showed that the first factor explained 19.57% of the total variance, which was below the commonly used threshold of 40%. Confirmatory factor analysis further showed that the theoretical four-factor model fit the data substantially better than the one-factor model. These findings suggested that serious common method bias was unlikely to substantially influence the results.

### 3.4 Indirect association through intentional self-regulation

**Table 3** summarizes the regression analyses for the indirect association model, with the key pathway coefficients presented in the main text. Fully adjusted results for all covariates are provided in **Supplementary Table 9**. All models included university fixed effects and were adjusted for demographic and academic covariates. Perceived school climate was negatively associated with employment anxiety (*B* = -0.205, *β* = -0.265, 95% *CI*: -0.229 to -0.181, *P* < 0.001) and positively associated with intentional self-regulation (*B* = 0.366, *β* = 0.254, 95% *CI*: 0.321 to 0.410, *P* < 0.001). When perceived school climate and intentional self-regulation were entered simultaneously, perceived school climate remained negatively associated with employment anxiety (*B* = -0.168, *β* = -0.217, 95% *CI*: -0.191 to -0.144, *P* < 0.001), and intentional self-regulation was also negatively associated with employment anxiety (*B* = -0.102, *β* = -0.189, 95% *CI*: -0.118 to -0.085, *P* < 0.001). The model accounted for 12.4% of the variance in employment anxiety (adjusted *R*²= 0.120), and the inclusion of intentional self-regulation increased the explained variance by 3.3% (Δ*R*²= 0.033).

**Table 3:** Summary of regression analyses for the mediation model.

Regression equation		Overall fit coefficient					Regression coefficient					
Outcome variables	Predictor variables	*R*	*R²*	Adjusted *R²*	Δ*R²*	*Robust F*	*β*	*B*	*HC3 SE*	*t*	95%*CI*	*P*
Employment anxiety	Perceived school climate	0.302	0.091	0.087	-	21.141	-0.265	-0.205	0.012	-16.833	(-0.229, -0.181)	<0.001
Intentional self-regulation	Perceived school climate	0.270	0.073	0.068	-	16.617	0.254	0.366	0.023	16.171	(0.321, 0.410)	<0.001
Employment anxiety	Perceived school climate	0.353	0.124	0.120	0.033	27.273	-0.217	-0.168	0.012	-13.707	(-0.191, -0.144)	<0.001
	Intentional self-regulation						-0.189	-0.102	0.008	-12.063	(-0.118, -0.085)	<0.001

Only the key predictors in the mediation paths are shown. Overall model significance was evaluated using HC3 robust Wald *F* tests, and coefficient-level inference was based on HC3 heteroskedasticity-consistent standard errors. All models included university fixed effects and were adjusted for age, sex, grade, residence, only-child status, family economic status, academic pressure, internship experience, and postgraduate entrance examination intention. Grade, family economic status, academic pressure, and university affiliation were entered as categorical variables using dummy coding.

**Table 4** presents the total, direct, and indirect associations of perceived school climate with employment anxiety through intentional self-regulation. Bootstrap analysis based on 5,000 resamples showed a statistically supported indirect association (*B* = -0.037, *SE* = 0.004, 95% bootstrap *CI*: -0.045 to -0.030). The completely standardized indirect effect was -0.048 (*SE* = 0.005, 95% bootstrap *CI*: -0.058 to -0.039). The direct association between perceived school climate and employment anxiety remained statistically significant after intentional self-regulation was included in the model (*B* = -0.168, *SE* = 0.012, 95% *CI*: -0.191 to -0.144). These findings support an indirect association through intentional self-regulation.

**Table 4:** Direct and indirect effects of perceived school climate on employment anxiety through intentional self-regulation.

Path	*B*	*SE*	95% *CI*
Total effect	-0.205	0.012	(-0.229, -0.181)
Direct effect	-0.168	0.012	(-0.191, -0.144)
Indirect effect	-0.037	0.004	(-0.045, -0.030)
Completely standardized indirect effect	−0.048	0.005	(−0.058, −0.039)

Bootstrap standard errors and confidence intervals were used for the indirect effects based on 5,000 resamples. Total and direct effects were estimated using HC3 robust standard errors.

### 3.5 Moderating role of perceived employability

**Figure 1** presents the estimated moderated mediation model, and **Table 5** summarizes the corresponding regression results. Fully adjusted estimates for all covariates are provided in **Supplementary Table 10**. All models included university fixed effects and were adjusted for demographic and academic covariates. In the main-effects model predicting employment anxiety, perceived school climate, intentional self-regulation, and perceived employability jointly explained 13.9% of the variance in employment anxiety (adjusted *R*² = 0.135). After the interaction term was added, perceived school climate (*B* = -0.133, *β* = -0.172, 95% CI: -0.158 to -0.108), intentional self-regulation (*B* = -0.103, *β* = -0.191, 95% *CI*: -0.119 to -0.087), and perceived employability (*B* = -0.126, *β* = -0.134, 95% *CI*: -0.156 to -0.096) remained negatively associated with employment anxiety (all *P* < 0.001). The interaction between intentional self-regulation and perceived employability was statistically significant (*B* = 0.005, *β* = 0.087, 95% *CI*: 0.003 to 0.006, *P* < 0.001). However, adding the interaction term increased the explained variance by only 0.8% (Δ*R*² = 0.008), and the final model explained 14.7% of the variance in employment anxiety (adjusted *R*² = 0.142). Thus, the moderating association was statistically detectable but small in magnitude.

**Figure 1:**
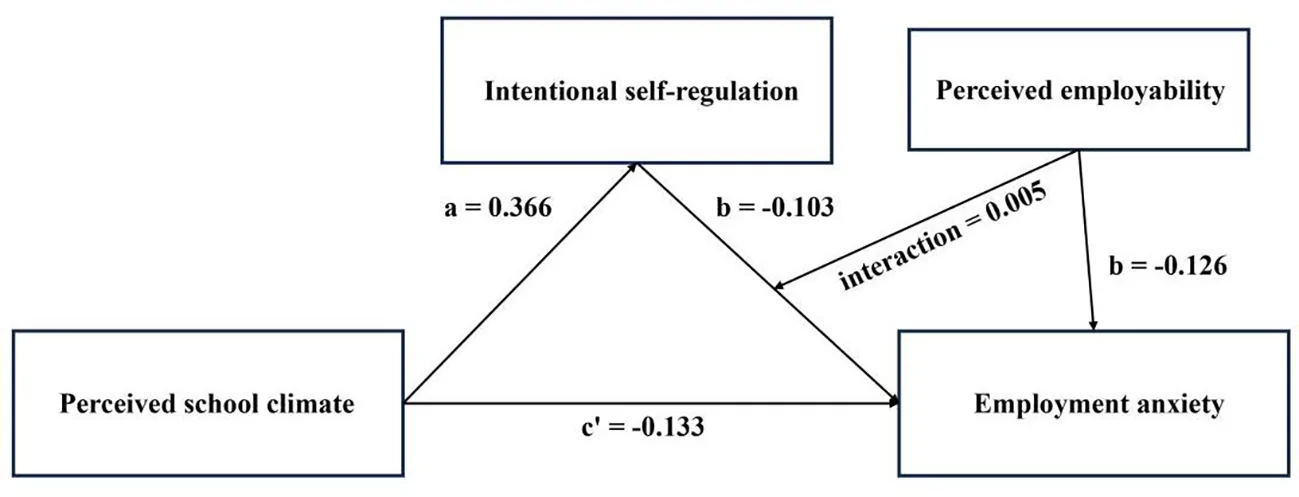
Estimated moderated mediation model linking perceived school climate, intentional self-regulation, perceived employability, and employment anxiety. Note. Coefficients are unstandardized regression estimates from the fully adjusted moderated mediation model. Intentional self-regulation and perceived employability were mean-centered before calculating the interaction term. All equations included university fixed effects and were adjusted for age, sex, grade, residence, only-child status, family economic status, academic pressure, internship experience, and postgraduate entrance examination intention.

**Table 5:** Summary of hierarchical regression analyses for the moderated mediation model.

Regression equation		Overall fit coefficient					Regression coefficient					
Outcome variables	Predictor variables	*R*	*R²*	Adjusted *R²*	Δ*R²*	*Robust F*	*β*	*B*	*HC3 SE*	*t*	95%*CI*	*P*
Intentional self-regulation	Perceived school climate	0.270	0.073	0.068	-	16.617	0.254	0.366	0.023	16.171	(0.321, 0.410)	<0.001
Employment anxiety	Perceived school climate	0.373	0.139	0.135	-	29.835						
Employment anxiety	Perceived school climate	0.383	0.147	0.142	0.008	30.995	-0.172	-0.133	0.013	-10.392	(-0.158, -0.108)	<0.001
	Intentional self-regulation						-0.191	-0.103	0.008	-12.440	(-0.119, -0.087)	<0.001
	Perceived employability						-0.134	-0.126	0.015	-8.240	(-0.156, -0.096)	<0.001
	Intentional self-regulation × perceived employability						0.087	0.005	0.001	5.790	(0.003, 0.006)	<0.001

Only the key predictors and the interaction term are shown. Overall model significance was evaluated using HC3 robust Wald *F* tests, and coefficient-level inference was based on HC3 heteroskedasticity-consistent standard errors. All models included university fixed effects and were adjusted for age, sex, grade, residence, only-child status, family economic status, academic pressure, internship experience, and postgraduate entrance examination intention. Grade, family economic status, academic pressure, and university affiliation were entered as categorical variables using dummy coding. Intentional self-regulation and perceived employability were mean-centered before calculating the interaction term.

**Figure 2** illustrates the interaction between intentional self-regulation and perceived employability in relation to employment anxiety. Intentional self-regulation was negatively associated with predicted employment anxiety at all three levels of perceived employability, but the association was stronger at lower perceived employability and weaker at higher perceived employability. Simple-slope analyses further showed that intentional self-regulation was negatively associated with employment anxiety at low (*B* = -0.150, 95% *CI*: -0.173 to -0.128), mean (*B* = -0.103, 95% *CI*: -0.119 to -0.087), and high levels of perceived employability (*B* = -0.055, 95% *CI*: -0.078 to -0.032; all *P* < 0.001). The magnitude of the negative association progressively decreased as perceived employability increased (**Supplementary Table 11**).

**Figure 2:**
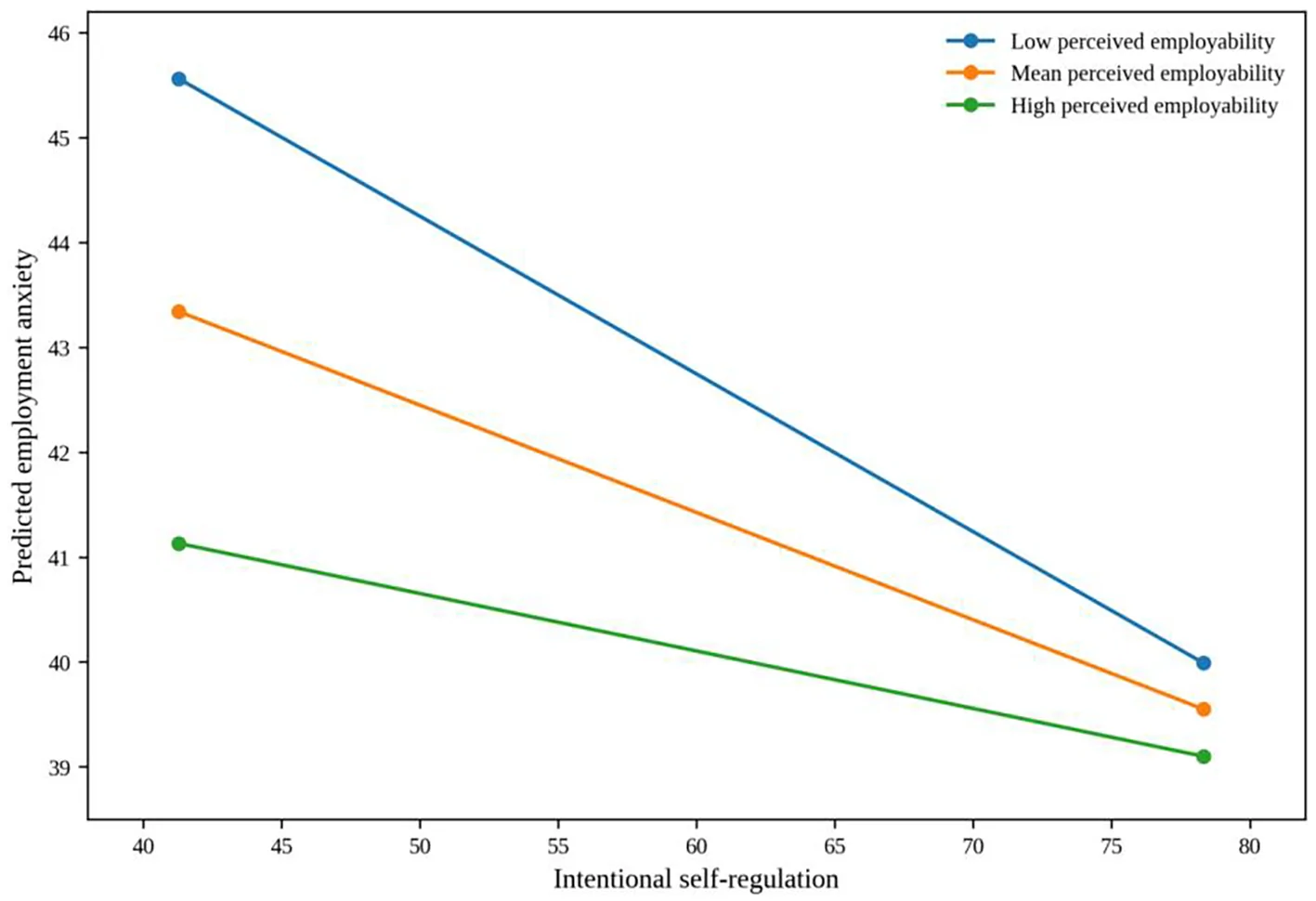
Moderating effect of perceived employability on the association between intentional self-regulation and employment anxiety. Predicted employment anxiety was estimated at low and high levels of intentional self-regulation and at low (−1 SD), mean, and high (+1 SD) levels of perceived employability. The model included university fixed effects and was adjusted for age, sex, grade, residence, only-child status, family economic status, academic pressure, internship experience, and postgraduate entrance examination intention.

**Figure 3** presents the conditional indirect associations of perceived school climate with employment anxiety through intentional self-regulation. The indirect association was −0.055 at low perceived employability (95% bootstrap CI: -0.070 to -0.045), -0.038 at the mean level (95% bootstrap CI: -0.049 to -0.031), and -0.020 at high perceived employability (95% bootstrap CI: -0.035 to -0.012). The index of moderated mediation was 0.002 (95% bootstrap CI: 0.001 to 0.002), indicating that the indirect association became progressively weaker as perceived employability increased.

**Figure 3:**
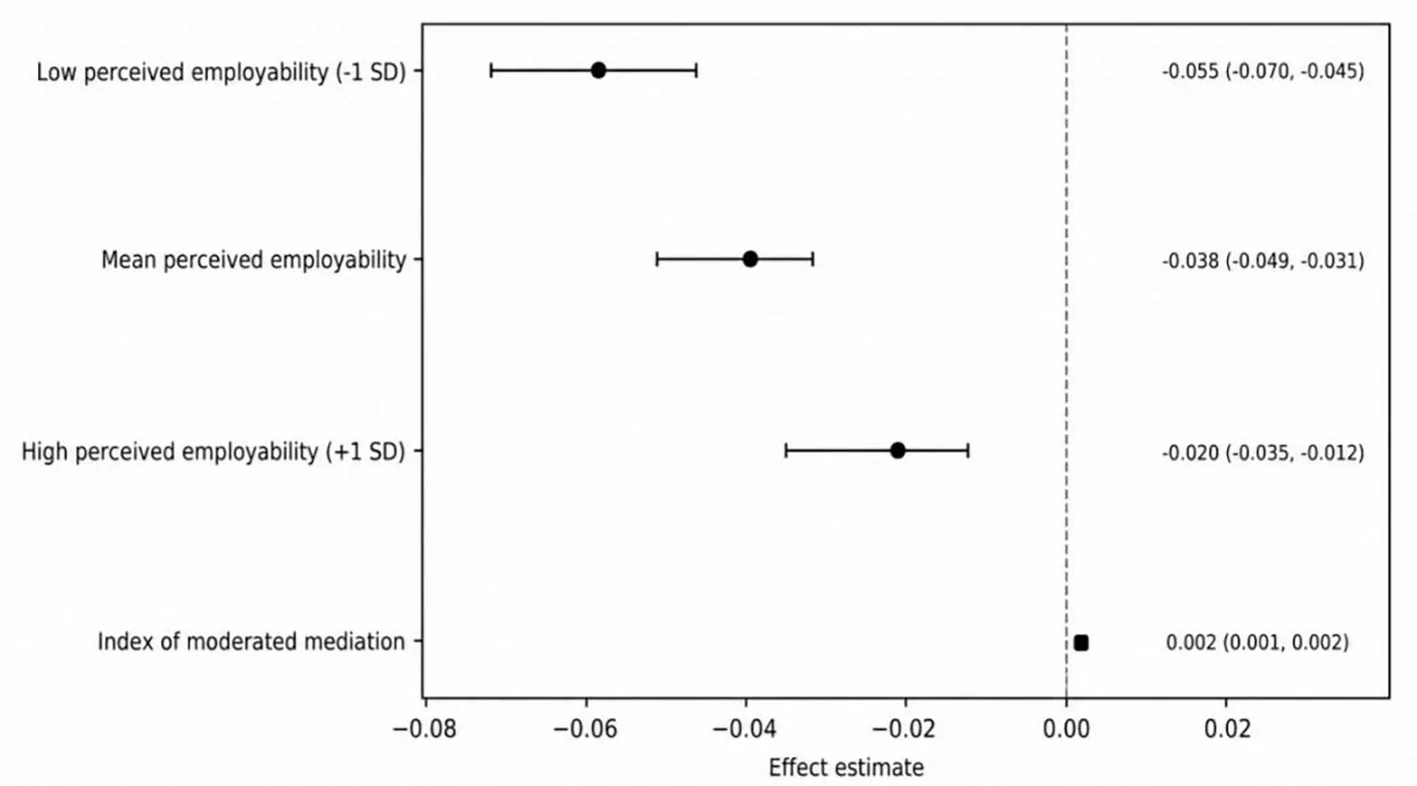
Conditional indirect effects across levels of perceived employability. Dots indicate conditional indirect effects or the index of moderated mediation, and horizontal lines indicate 95% bootstrap confidence intervals. Bootstrap estimates were based on 5,000 resamples. The estimates were derived from the fully adjusted model, which included university fixed effects and the same covariates as the primary analysis.

### 3.6 Sensitivity and subgroup analyses

Leave-one-university-out sensitivity analyses showed that the principal findings remained stable after each university was sequentially excluded. The indirect associations ranged from -0.034 to -0.041, and all corresponding bootstrap confidence intervals excluded zero. The interaction coefficients ranged from 0.004 to 0.005 and remained statistically significant in all analyses (all *P* < 0.001), indicating that the findings were not driven by any single university (**Supplementary Table 12**). The inverse association between perceived school climate and employment anxiety was also generally consistent across the examined demographic and academic subgroups, with no statistically significant interaction detected for any subgroup-defining variable (**Supplementary Figure 2**).

## 4 Discussion

This study extends previous research by integrating perceived school climate, intentional self-regulation, and perceived employability within a single conditional process framework for employment anxiety among medical undergraduates. A more positive perceived school climate was associated with lower employment anxiety both directly and indirectly through intentional self-regulation, whereas perceived employability modestly conditioned the association between intentional self-regulation and employment anxiety. These findings identify a theoretically coherent pattern in which a relatively distal educational context, an individual self-regulatory process, and a career-specific appraisal are jointly related to psychological adaptation during the school-to-work transition.

The negative association between perceived school climate and employment anxiety is consistent with the view that educational contexts are closely related to students’ psychological adaptation. In this study, perceived school climate included teacher support, peer support, fairness, belonging, psychological support, and career-development resources. These components may represent contextual resources that are relevant to students’ appraisal of future uncertainty. A more positive school climate may be associated with lower employment anxiety because students who perceive greater support and belonging may feel less isolated when facing career-related pressure. This finding also aligns with recent evidence linking school or campus climate with student mental health and well-being. Studies have shown that positive school climate is associated with better mental well-being, while school support and school belonging are related to lower psychological distress (23, 24). Evidence from college settings further suggests that students’ perceptions of the campus mental health climate are associated with psychological distress and help-seeking intentions (25). For medical undergraduates, this association may be particularly relevant because they face intensive academic demands, clinical training pressures, and uncertainty regarding postgraduate education, residency training, specialty selection, and geographic mobility (26). Perceived school climate reflects students’ appraisal of their educational environment and may therefore be influenced by individual affective processes. Students with greater employment anxiety may evaluate institutional support and interpersonal relationships less favorably, while unmeasured factors such as negative affectivity or previous psychological distress may influence both perceptions. Although university fixed effects addressed average institutional differences, individual-level perceptual influences remain possible. Accordingly, perceived school climate may reflect both institutional conditions and students’ psychological appraisal of those conditions.

The indirect association through intentional self-regulation provides a plausible explanation for how educational context may be translated into responses to career uncertainty. Intentional self-regulation involves goal selection, resource optimization, strategy adjustment, and compensation when facing barriers. These processes are relevant to employment preparation because medical undergraduates need to evaluate career options, organize information, mobilize institutional resources, and revise plans when preferred pathways are unavailable. Recent evidence indicates that intentional self-regulation enables young people to seek and use environmental resources and is associated with positive developmental outcomes (27). Self-regulation has also been related to psychological adjustment and the perceived need for psychological help (28). In employment-related contexts, psychological resources and active coping have been associated with employment expectations (29), while social and personal resources have been linked to lower job-search anxiety and greater flourishing (30). These findings are consistent with relational developmental systems theory, in which adaptive outcomes arise through the alignment of contextual resources with individual strengths. Intentional self-regulation may help explain how educational support is associated with lower employment anxiety, but the indirect association was modest and a substantial direct association remained. Other processes, including belonging, social support, career self-efficacy, professional identity, and access to employment information, may also be involved.

The moderation findings were broadly consistent with a compensatory-resource interpretation. The negative association between intentional self-regulation and employment anxiety was strongest at low perceived employability and progressively weaker at average and high perceived employability. Students who doubt their employment competence or future job prospects may depend more heavily on goal clarification, adaptive planning, resource mobilization, and alternative strategy development to manage employment uncertainty. By contrast, students with higher perceived employability may already experience greater confidence and perceived control, reducing the relative contribution of self-regulatory capacity. This interpretation is consistent with evidence linking self-perceived employability with student well-being and institutional embeddedness (31), as well as longitudinal findings connecting university-related resources with perceived employability and mental well-being (32). Career adaptability and related career resources have also been associated with graduate mental health, job-search behavior, and subjective well-being (33, 34). However, the interaction explained only an additional 0.8% of the variance in employment anxiety, indicating a modest moderation pattern with limited incremental explanatory value. This pattern should also be understood within the context of medical education in Heilongjiang Province, where perceived employability may be shaped by regional labor demand, postgraduate and residency pathways, and expectations regarding geographic mobility.

These findings support a coordinated approach to employment-related psychological support in medical universities. At the institutional level, universities could strengthen teacher and peer support, procedural fairness, belonging, accessible psychological services, and transparent information about postgraduate training and employment pathways. Career education could incorporate structured goal setting, adaptive planning, resource seeking, obstacle management, and alternative career planning. Students reporting low perceived employability, elevated employment anxiety, or difficulty managing career goals may benefit from more targeted counselling that integrates career guidance with psychological support.

Several limitations warrant consideration. The cross-sectional design precluded causal or temporal inference, and reverse causality remains possible; accordingly, the indirect effects should be interpreted as statistical associations rather than evidence of longitudinal mediation. All variables were self-reported, which may have introduced recall, social desirability, and common method bias, although no single common factor dominated the covariance structure. Relatedly, measurement-related concerns also remain. Although the perceived school climate total-score model showed good fit and high reliability, the Delaware School Climate Survey was developed for K-12 settings, and its average variance extracted was below 0.50. Its convergent validity in medical undergraduates should therefore be interpreted cautiously, and the findings do not constitute full validation of its original multidimensional structure in higher education. Residual confounding cannot be excluded because personality, prior psychological distress, academic achievement, professional identity, career self-efficacy, family expectations, and access to career resources were not measured. Internship experience was assessed only as a binary variable, without information on duration, timing, intensity, quality, specialty, or setting. The modest indirect and interaction associations also indicate that substantial variation in employment anxiety remains unexplained. Moreover, although university fixed effects and leave-one-university-out analyses addressed some heterogeneity, only four universities were included, limiting multilevel assessment of institutional characteristics. All institutions were located in Heilongjiang Province, which may restrict generalizability across regions with different labor markets and educational systems. Future studies should use longitudinal, multiregional, and multilevel designs, repeated measurements, objective employment indicators, and more detailed assessments of clinical training and career-related resources.

## 5 Conclusion

Positive perceived school climate was associated with lower employment anxiety partly through intentional self-regulation, while perceived employability modestly conditioned this pathway. By integrating educational context, self-regulatory capacity, and career-specific appraisal within a single conditional process model, this study clarifies how and for whom school climate is related to employment anxiety during the school-to-work transition. The findings suggest that medical universities should combine school-climate improvement with self-regulation training and provide more targeted career counselling and employment support for students with low perceived employability.

## Data Availability

The datasets are not publicly available due to ethical and privacy restrictions. De-identified data may be available upon reasonable request, subject to ethics approval and institutional permissions.
