# Supplementary material for: Association between perceived school climate and employment anxiety among medical undergraduates: the mediating role of intentional self-regulation and the moderating role of perceived employability
Source: Front Psychiatry. 2026 Sep 15;17:1927113. doi: 10.3389/fpsyt.2026.1927113 (PMC13619818; doi:10.3389/fpsyt.2026.1927113)
Supplement: Supplementary file 1 [file DataSheet1.pdf]

Supplementary Material

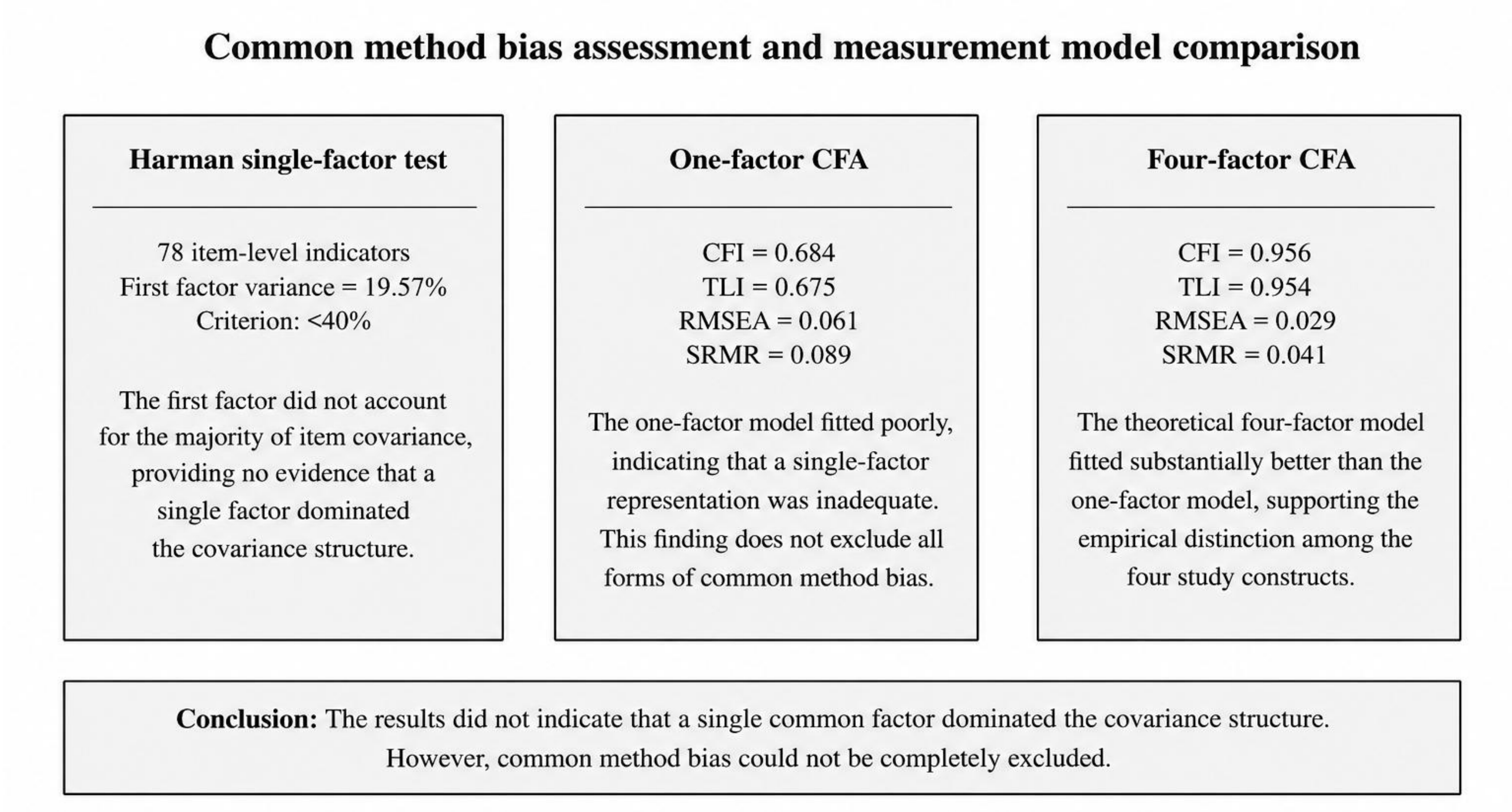

Supplementary Figure S1. Common method bias assessment and measurement model comparison.

Note. All 78 item-level indicators from the four scales were entered directly; item parcels were not used. CFA, confirmatory factor analysis; CFI, comparative fit index; TLI, Tucker–Lewis index; RMSEA, root mean square error of approximation; SRMR, standardized root mean square residual.

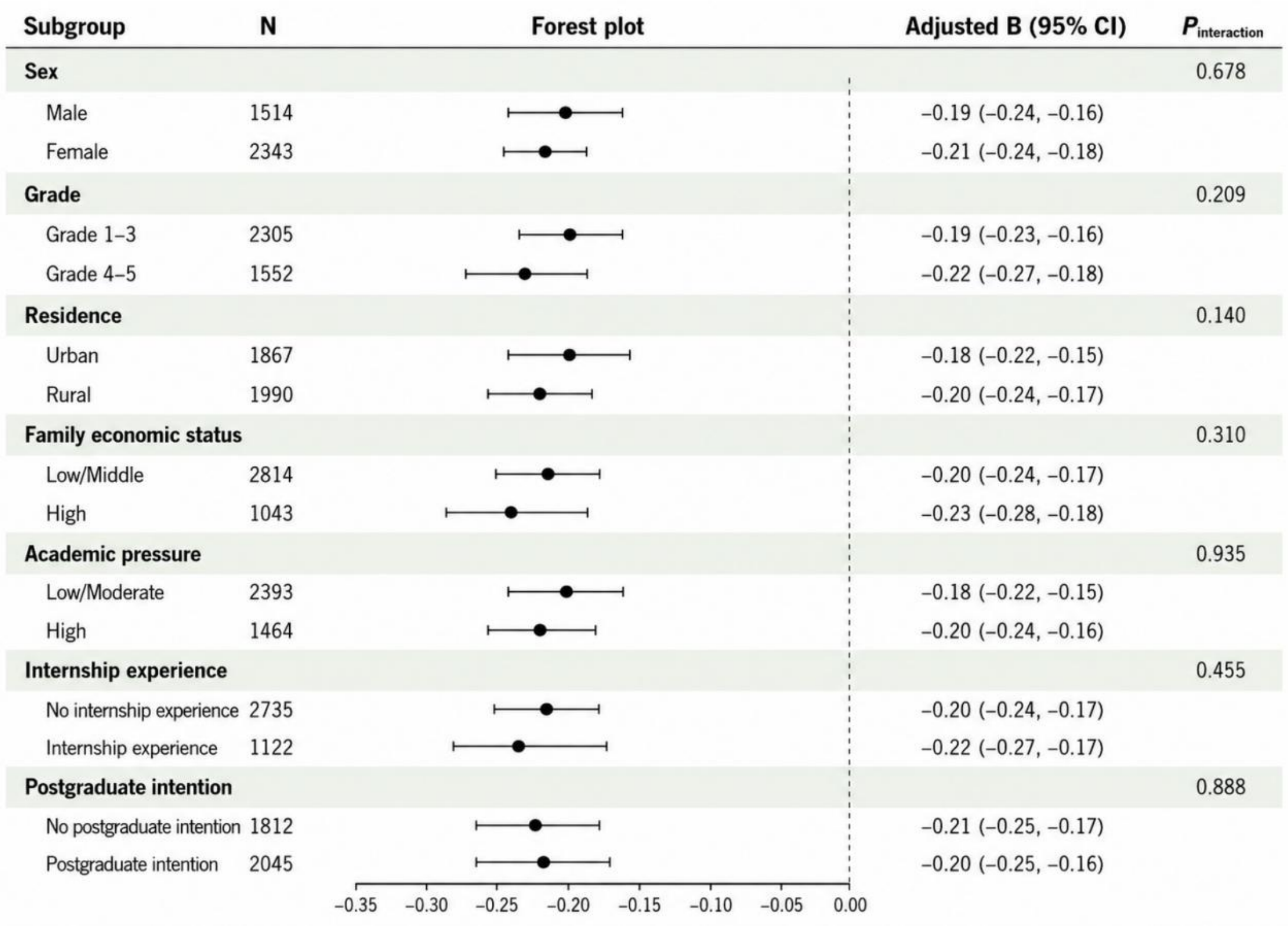

Supplementary Figure S2. Subgroup analysis of the association between perceived school climate and employment anxiety

Note. Dots indicate adjusted regression coefficients, and horizontal lines indicate 95% confidence intervals. Pinteraction values are presented for each subgroup-defining variable. Each subgroup model retained university fixed effects and was adjusted for the remaining covariates where applicable.

Supplementary Table S1. Comparison of one-factor and theoretically multidimensional CFA models for the four scales.

Scale	Model	χ^2	*df*	*CFI*	*TLI*	*RMSEA*	*SRMR*	Interfactor r range
Perceived school climate	One-factor total-score model	730.007	405	0.994	0.993	0.014	0.012	
	Seven correlated content factors	721.009	384	0.993	0.993	0.015	0.012	0.980–1.000
Intentional self-regulation	One-factor total-score model	237.914	135	0.997	0.997	0.014	0.010	
	Three correlated SOC factors	236.935	132	0.997	0.997	0.014	0.010	0.996–1.000
Perceived employability	One-factor total-score model	162.294	104	0.998	0.998	0.012	0.010	
	Two correlated internal/external factors	158.615	103	0.998	0.998	0.012	0.010	0.993
Employment anxiety	One-factor total-score model	108.744	77	0.999	0.999	0.010	0.009	
	Two correlated symptom factors	108.744	76	0.999	0.999	0.011	0.009	1.000

Note. Item-level CFA was based on polychoric correlation matrices. Item-level CFA was conducted using the weighted least squares mean- and variance-adjusted estimator, with items specified as ordered categorical indicators. Robust model-fit indices are reported. The multidimensional models followed the item groupings specified in the questionnaire: seven school-climate content factors, three selection–optimization–compensation factors, two internal/external employability factors, and two employment-anxiety symptom factors. The multidimensional models did not materially improve global fit, and interfactor correlations approached unity. Accordingly, total scores were retained, and the results should not be interpreted as validating distinct subscale scores. CFI, comparative fit index; TLI, Tucker–Lewis index; RMSEA, root mean square error of approximation; SRMR, standardized root mean square residual.

Supplementary Table S2. Reliability and convergent validity of the four scales.

Scale	Items	Cronbach α	Ordinal α	McDonald's ω	*AVE*	Loading range	Corrected item–total r range
Perceived school climate	30	0.941	0.956	0.956	0.421	0.622–0.674	0.547–0.592
Intentional self-regulation	18	0.930	0.945	0.945	0.489	0.680–0.720	0.608–0.644
Perceived employability	16	0.925	0.937	0.937	0.482	0.679–0.708	0.620–0.647
Employment anxiety	14	0.920	0.932	0.932	0.495	0.676–0.726	0.617–0.664

Note. B9, B14, and B26 were reverse-scored, and B31 was excluded because it was an attention-check item. Ordinal α and McDonald's ω were calculated from the polychoric-correlation one-factor models. Reliability was high for all four scales. AVE values ranged from 0.421 to 0.495 and were below the conventional 0.50 benchmark; convergent validity should therefore be described as borderline and interpreted cautiously rather than as fully established. AVE, average variance extracted.

Supplementary Table S3. Regression diagnostics for the final moderated mediation outcome model.

Diagnostic	Result	Interpretation
VIF for key predictors	1.006–1.221	No evidence of multicollinearity among the focal predictors
Maximum VIF	5.173	Observed for the fourth-year grade dummy; below the common threshold of 10
Breusch–Pagan test	LM = 13.886, P = 0.874	No evidence of heteroscedasticity
Ramsey RESET	F = 0.238, P = 0.625	No evidence of major functional-form misspecification
Residual skewness	-0.010	Approximately symmetric residual distribution
Residual excess kurtosis	-0.064	Approximately normal tail behavior

Note. HC3 robust standard errors were retained for all final models as a conservative specification despite the nonsignificant Breusch-Pagan test.

Supplementary Table S4. Discriminant validity of the four study constructs.

Construct	*PSC*	*ISR*	*PE*	*EA*
Perceived school climate	0.649	0.278	0.363	0.298
Intentional self-regulation	0.260	0.700	0.078	0.270
Perceived employability	0.339	0.073	0.695	0.229
Employment anxiety	-0.277	-0.250	-0.211	0.704

Note. Square roots of AVE are shown on the diagonal, construct-score correlations are shown below the diagonal, and heterotrait–monotrait ratios are shown above the diagonal. Each square root of AVE exceeded the absolute correlations involving the corresponding construct, and all HTMT values were below 0.85, supporting construct-level discriminant validity. Negative correlations involving employment anxiety reflect the expected inverse direction. AVE, average variance extracted; HTMT, heterotrait–monotrait ratio; PSC, perceived school climate; ISR, intentional self-regulation; PE, perceived employability; EA, employment anxiety.

Supplementary Table S5. Standardized one-factor loadings and corrected item–total correlations for all four scales.

Scale	Item	Standardized loading	Corrected item–total r
Perceived school climate			
	B1	0.650	0.571
	B2	0.652	0.573
	B3	0.667	0.585
	B4	0.643	0.566
	B5	0.648	0.570
	B6	0.672	0.592
	B7	0.674	0.590
	B8	0.633	0.558
	B9	0.654	0.576
	B10	0.643	0.563
	B11	0.672	0.591
	B12	0.640	0.564
	B13	0.622	0.547
	B14	0.662	0.583
	B15	0.660	0.580
	B16	0.641	0.563
	B17	0.645	0.567
	B18	0.636	0.560
	B19	0.632	0.555
	B20	0.637	0.560
	B21	0.646	0.570
	B22	0.641	0.562
	B23	0.632	0.556
	B24	0.647	0.571
	B25	0.644	0.567
	B26	0.640	0.564
	B27	0.663	0.585
	B28	0.664	0.586
	B29	0.644	0.568
	B30	0.653	0.575
Intentional self-regulation			
	C1	0.702	0.626
	C2	0.688	0.615
	C3	0.703	0.631
	C4	0.720	0.644

Scale	Item	Standardized loading	Corrected item–total r
	C5	0.703	0.628
	C6	0.698	0.622
	C7	0.701	0.627
	C8	0.703	0.632
	C9	0.706	0.631
	C10	0.690	0.619
	C11	0.702	0.629
	C12	0.680	0.608
	C13	0.702	0.630
	C14	0.700	0.624
	C15	0.686	0.612
	C16	0.703	0.631
	C17	0.706	0.631
	C18	0.700	0.629
Perceived employability			
	D1	0.690	0.630
	D2	0.683	0.624
	D3	0.708	0.647
	D4	0.701	0.639
	D5	0.683	0.624
	D6	0.704	0.643
	D7	0.686	0.626
	D8	0.696	0.634
	D9	0.691	0.631
	D10	0.702	0.640
	D11	0.701	0.639
	D12	0.701	0.641
	D13	0.704	0.643
	D14	0.689	0.629
	D15	0.679	0.620
	D16	0.695	0.635
Employment anxiety			
	E1	0.708	0.647
	E2	0.721	0.659
	E3	0.726	0.664
	E4	0.705	0.644
	E5	0.676	0.617

Scale	Item	Standardized loading	Corrected item–total r
	E6	0.706	0.645
	E7	0.692	0.633
	E8	0.704	0.643
	E9	0.702	0.641
	E10	0.695	0.636
	E11	0.698	0.639
	E12	0.700	0.640
	E13	0.705	0.644
	E14	0.713	0.651

Note. Standardized loadings were obtained from the one-factor polychoric-correlation CFA models. All loadings and corrected item–total correlations were positive. These item-level results support use of the total scores but do not establish the distinctness of the original subdimensions.

Supplementary Table S6. Post-hoc comparisons following significant omnibus ANOVA tests.

Variable	Mean difference	95% *CI*	Adjusted *P*
Grade			
Second-year vs First-year	0.411	(-1.102, 1.923)	0.947
Third-year vs First-year	-0.856	(-2.294, 0.582)	0.481
Fourth-year vs First-year	-0.867	(-2.303, 0.568)	0.466
Fifth-year vs First-year	-0.080	(-1.706, 1.547)	1.000
Third-year vs Second-year	-1.267	(-2.577, 0.043)	0.064
Fourth-year vs Second-year	-1.278	(-2.585, 0.029)	0.059
Fifth-year vs Second-year	-0.490	(-2.005, 1.024)	0.903
Fourth-year vs Third-year	-0.011	(-1.231, 1.208)	1.000
Fifth-year vs Third-year	0.777	(-0.663, 2.216)	0.581
Fifth-year vs Fourth-year	0.788	(-0.649, 2.225)	0.565
Family economic status			
Low vs High	1.398	(0.371, 2.425)	0.004
Middle vs High	0.396	(-0.511, 1.304)	0.562
Middle vs Low	-1.002	(-1.916, -0.087)	0.028
Academic pressure			
Low vs High	-2.703	(-3.681, -1.724)	<0.001
Moderate vs High	-1.741	(-2.596, -0.885)	<0.001
Moderate vs Low	0.962	(-0.015, 1.939)	0.055
University			
University B vs University A	0.566	(-0.593, 1.724)	0.592
University C vs University A	-0.101	(-1.280, 1.078)	0.996
University D vs University A	1.168	(-0.017, 2.353)	0.055
University C vs University B	-0.666	(-1.812, 0.479)	0.440
University D vs University B	0.602	(-0.549, 1.754)	0.535
University D vs University C	1.269	(0.097, 2.441)	0.028

Note. Grade showed a significant omnibus ANOVA result, but no pairwise comparison remained significant after Tukey adjustment. Low family economic status was associated with higher employment anxiety than middle and high family economic status. High academic pressure was associated with higher employment anxiety than low and moderate academic pressure. University D had higher employment anxiety than University C after adjustment for multiple comparisons.

Supplementary Table S7. University-specific distributions of the main study variables.

Variable	University A (n = 921)	University B (n = 1034)	University C (n = 961)	University D (n = 941)	*F*	*P*
Perceived school climate	89.96 ± 12.91	90.56 ± 12.83	89.59 ± 12.63	89.64 ± 13.16	1.216	0.302
Intentional self-regulation	60.16 ± 18.88	60.59 ± 18.30	58.83 ± 19.10	59.56 ± 17.75	1.679	0.169
Perceived employability	51.40 ± 10.77	51.92 ± 10.64	52.22 ± 9.89	51.69 ± 10.94	1.013	0.386
Employment anxiety	41.10 ± 10.30	41.66 ± 9.63	41.00 ± 9.96	42.27 ± 9.92	3.273	0.020

Supplementary Table S8. Results of common method bias assessment.

Test	Analytic strategy	Key result	Fit indices or criteria	Interpretation
Harman single-factor test	All 78 item-level indicators were entered into an unrotated exploratory factor analysis.	Four factors had eigenvalues greater than 1. The first factor explained 19.57% of the total variance.	First factor variance < 40%.	The first factor did not account for the majority of item covariance, providing no evidence that a single factor dominated the covariance structure.
One-factor CFA	All 78 items were specified to load on one latent factor.	The one-factor model showed poor overall fit.	CFI = 0.684; TLI = 0.675; RMSEA = 0.061; SRMR = 0.089.	The one-factor model fitted poorly, indicating that asingle-factor representation was inadequate. This findingdoes not exclude all forms of common method bias.
Four-factor CFA comparison	Items were specified according to the theoretical constructs: perceived school climate, intentional self-regulation, perceived employability, and employment anxiety.	The theoretical four-factor model showed substantially better fit.	CFI = 0.956; TLI = 0.954; RMSEA = 0.029; SRMR = 0.041.	The theoretical four-factor model fitted substantially better than the one-factor model, supporting construct distinction.

Note. CFA, confirmatory factor analysis; CFI, comparative fit index; TLI, Tucker Lewis index; RMSEA, root mean square error of approximation; SRMR, standardized root mean square residual.

Supplementary Table S9. Fully adjusted hierarchical regression results for the mediation model.

Regression equation		Overall fit coefficient			Regression coefficient					
Outcome variables	Predictor variables	*R*	*R²*	*Robust F*	*β*	*B*	HC3 *SE*	*t*	95% *CI*	*P*
Employment anxiety	Perceived school climate	0.302	0.091	21.141	-0.265	-0.205	0.012	-16.833	(-0.229, -0.181)	<0.001
	Age				0.045	0.297	0.188	1.577	(-0.072, 0.666)	0.115
	Sex (reference: Male)									
	Female				-0.009	-0.173	0.315	-0.550	(-0.792, 0.445)	0.582
	Grade (reference: First-year)									
	Second-year				0.011	0.274	0.548	0.500	(-0.800, 1.349)	0.617
	Third-year				-0.053	-1.201	0.616	-1.949	(-2.409, 0.007)	0.051
	Fourth-year				-0.065	-1.468	0.799	-1.838	(-3.034, 0.097)	0.066
	Fifth-year				-0.038	-1.071	0.980	-1.093	(-2.992, 0.849)	0.274
	Residence (reference: Rural)									
	Urban				-0.010	-0.193	0.308	-0.627	(-0.795, 0.410)	0.531
	Only-child status (reference: No)									
	Yes				0.027	0.551	0.317	1.740	(-0.070, 1.172)	0.082
	Family economic status (reference: Middle)									
	Low				0.026	0.581	0.374	1.553	(-0.152, 1.314)	0.120
	High				-0.005	-0.113	0.375	-0.301	(-0.848, 0.623)	0.763
	Academic pressure (reference: Moderate)									
	Low				-0.035	-0.811	0.403	-2.014	(-1.601, -0.022)	0.044
	High				0.064	1.312	0.351	3.732	(0.623, 2.000)	<0.001
	Internship experience (reference: No)									
	Yes				-0.003	-0.059	0.429	-0.138	(-0.899, 0.781)	0.891
	Postgraduate entrance examination intention (reference: No)									

Regression equation		Overall fit coefficient			Regression coefficient					
	Yes				-0.014	-0.282	0.307	-0.918	(-0.885, 0.320)	0.359
	University (reference: University A)									
	University B				0.033	0.742	0.434	1.711	(-0.108, 1.592)	0.087
	University C				-0.006	-0.142	0.450	-0.316	(-1.025, 0.740)	0.752
	University D				0.050	1.161	0.450	2.581	(0.279, 2.042)	0.010
Intentional self-regulation	Perceived school climate	0.270	0.073	16.617	0.254	0.366	0.023	16.171	(0.321, 0.410)	<0.001
	Age				0.026	0.318	0.353	0.904	(-0.371, 1.007)	0.366
	Sex (reference: Male)									
	Female				0.001	0.045	0.594	0.076	(-1.118, 1.209)	0.939
	Grade (reference: First-year)									
	Second-year				-0.039	-1.817	1.074	-1.692	(-3.921, 0.288)	0.091
	Third-year				-0.030	-1.255	1.190	-1.055	(-3.587, 1.077)	0.291
	Fourth-year				-0.035	-1.493	1.475	-1.012	(-4.385, 1.399)	0.312
	Fifth-year				-0.031	-1.635	1.831	-0.893	(-5.224, 1.953)	0.372
	Residence (reference: Rural)									
	Urban				0.009	0.317	0.578	0.549	(-0.815, 1.449)	0.583
	Only-child status (reference: No)									
	Yes				-0.024	-0.910	0.590	-1.543	(-2.066, 0.246)	0.123
	Family economic status (reference: Middle)									
	Low				-0.034	-1.434	0.705	-2.035	(-2.816, -0.053)	0.042
	High				0.010	0.396	0.700	0.567	(-0.975, 1.768)	0.571
	Academic pressure (reference: Moderate)									
	Low				0.003	0.152	0.756	0.201	(-1.330, 1.634)	0.841
	High				-0.003	-0.130	0.664	-0.195	(-1.431, 1.172)	0.845

Regression equation		Overall fit coefficient			Regression coefficient					
	Internship experience (reference: No)									
	Yes				-0.008	-0.322	0.791	-0.407	(-1.872, 1.228)	0.684
	Postgraduate entrance examination intention (reference: No)									
	Yes				0.035	1.299	0.579	2.244	(0.164, 2.434)	0.025
	University (reference: University A)									
	University B				0.005	0.197	0.811	0.243	(-1.393, 1.787)	0.808
	University C				-0.030	-1.263	0.849	-1.489	(-2.927, 0.400)	0.137
	University D				-0.011	-0.468	0.823	-0.568	(-2.080, 1.145)	0.570
Employment anxiety	Perceived school climate	0.353	0.124	27.273	-0.217	-0.168	0.012	-13.707	(-0.191, -0.144)	<0.001
	Intentional self-regulation				-0.189	-0.102	0.008	-12.063	(-0.118, -0.085)	<0.001
	Age				0.050	0.329	0.185	1.784	(-0.033, 0.691)	0.074
	Sex (reference: Male)									
	Female				-0.008	-0.169	0.309	-0.546	(-0.775, 0.437)	0.585
	Grade (reference: First-year)									
	Second-year				0.004	0.089	0.542	0.165	(-0.972, 1.151)	0.869
	Third-year				-0.058	-1.329	0.606	-2.193	(-2.517, -0.141)	0.028
	Fourth-year				-0.071	-1.620	0.786	-2.060	(-3.161, -0.079)	0.039
	Fifth-year				-0.044	-1.238	0.958	-1.292	(-3.115, 0.639)	0.196
	Residence (reference: Rural)									
	Urban				-0.008	-0.160	0.302	-0.531	(-0.752, 0.432)	0.595
	Only-child status (reference: No)									
	Yes				0.022	0.459	0.311	1.476	(-0.150, 1.068)	0.140
	Family economic status (reference: Middle)									
	Low				0.019	0.435	0.370	1.178	(-0.289, 1.160)	0.239

Regression equation	Overall fit coefficient	Regression coefficient					
High		-0.003	-0.073	0.367	-0.198	(-0.792, 0.647)	0.843
Academic pressure (reference: Moderate)							
Low		-0.034	-0.796	0.395	-2.013	(-1.571, -0.021)	0.044
High		0.063	1.298	0.345	3.766	(0.623, 1.974)	<0.001
Internship experience (reference: No)							
Yes		-0.004	-0.092	0.420	-0.218	(-0.916, 0.732)	0.827
Postgraduate entrance examination intention (reference: No)							
Yes		-0.008	-0.150	0.302	-0.497	(-0.743, 0.442)	0.619
University (reference: University A)							
University B		0.034	0.762	0.427	1.783	(-0.076, 1.599)	0.075
University C		-0.012	-0.271	0.440	-0.615	(-1.134, 0.592)	0.539
University D		0.048	1.113	0.440	2.528	(0.250, 1.976)	0.011

Note. Grade, family economic status, academic pressure, and university affiliation were treated as categorical variables and entered using dummy coding. University affiliation was included as a fixed effect in all models. Overall model significance was evaluated using HC3 robust Wald F tests, and coefficient-level inference was based on HC3 heteroskedasticity-consistent standard errors.

Supplementary Table S10. Fully adjusted hierarchical regression results for the moderated mediation model.

	Regression equation	Overall fit coefficient			Regression coefficient					
**Outcome variables**	**Predictor variables**	***R***	***R²***	***Robust F***	***β***	***B***	**HC3 *SE***	***t***	**95% *CI***	***P***
Intentional self-regulation	Mediator model	0.270	0.073	16.617						
	Perceived school climate				0.254	0.366	0.023	16.171	(0.321, 0.410)	<0.001
	Age				0.026	0.318	0.353	0.904	(-0.371, 1.007)	0.366
	Sex (reference: Male)									
	Female				0.001	0.045	0.594	0.076	(-1.118, 1.209)	0.939
	Grade (reference: First-year)									
	Second-year				-0.039	-1.817	1.074	-1.692	(-3.921, 0.288)	0.091
	Third-year				-0.030	-1.255	1.190	-1.055	(-3.587, 1.077)	0.291
	Fourth-year				-0.035	-1.493	1.475	-1.012	(-4.385, 1.399)	0.312
	Fifth-year				-0.031	-1.635	1.831	-0.893	(-5.224, 1.953)	0.372
	Residence (reference: Rural)									
	Urban				0.009	0.317	0.578	0.549	(-0.815, 1.449)	0.583
	Only-child status (reference: No)									
	Yes				-0.024	-0.910	0.590	-1.543	(-2.066, 0.246)	0.123
	Family economic status (reference: Middle)									
	Low				-0.034	-1.434	0.705	-2.035	(-2.816, -0.053)	0.042
	High				0.010	0.396	0.700	0.567	(-0.975, 1.768)	0.571
	Academic pressure (reference: Moderate)									
	Low				0.003	0.152	0.756	0.201	(-1.330, 1.634)	0.841
	High				-0.003	-0.130	0.664	-0.195	(-1.431, 1.172)	0.845
	Internship experience (reference: No)									
	Yes				-0.008	-0.322	0.791	-0.407	(-1.872, 1.228)	0.684
	Postgraduate entrance examination intention (reference: No)									

	Regression equation	Overall fit coefficient			Regression coefficient					
	Yes				0.035	1.299	0.579	2.244	(0.164, 2.434)	0.025
	University (reference: University A)									
	University B				0.005	0.197	0.811	0.243	(-1.393, 1.787)	0.808
	University C				-0.030	-1.263	0.849	-1.489	(-2.927, 0.400)	0.137
	University D				-0.011	-0.468	0.823	-0.568	(-2.080, 1.145)	0.570
Employment anxiety	Main-effects model	0.373	0.139	29.835						
	Perceived school climate				-0.174	-0.134	0.013	-10.502	(-0.159, -0.109)	<0.001
	Intentional self-regulation				-0.192	-0.103	0.008	-12.363	(-0.119, -0.087)	<0.001
	Perceived employability				-0.130	-0.123	0.015	-7.957	(-0.153, -0.093)	<0.001
Employment anxiety	Interaction model	0.383	0.147	30.995						
	Perceived school climate				-0.172	-0.133	0.013	-10.392	(-0.158, -0.108)	<0.001
	Intentional self-regulation				-0.191	-0.103	0.008	-12.440	(-0.119, -0.087)	<0.001
	Perceived employability				-0.134	-0.126	0.015	-8.240	(-0.156, -0.096)	<0.001
	Intentional self-regulation × perceived employability				0.087	0.005	0.001	5.790	(0.003, 0.006)	<0.001
	Age				0.050	0.327	0.183	1.789	(-0.031, 0.686)	0.074
	Sex (reference: Male)									
	Female				-0.004	-0.076	0.306	-0.250	(-0.675, 0.523)	0.803
	Grade (reference: First-year)									
	Second-year				0.002	0.055	0.538	0.102	(-1.000, 1.110)	0.919
	Third-year				-0.060	-1.364	0.601	-2.268	(-2.543, -0.185)	0.023
	Fourth-year				-0.070	-1.587	0.782	-2.029	(-3.121, -0.054)	0.042
	Fifth-year				-0.040	-1.135	0.949	-1.195	(-2.995, 0.726)	0.232
	Residence (reference: Rural)									
	Urban				-0.003	-0.068	0.298	-0.227	(-0.653, 0.517)	0.820

Regression equation	Overall fit coefficient		Regression coefficient				
Only-child status (reference: No)							
Yes		0.022	0.452	0.307	1.471	(-0.150, 1.053)	0.141
Family economic status (reference: Middle)							
Low		0.012	0.265	0.365	0.724	(-0.452, 0.981)	0.469
High		-0.004	-0.084	0.363	-0.232	(-0.795, 0.627)	0.817
Academic pressure (reference: Moderate)							
Low		-0.031	-0.732	0.391	-1.873	(-1.498, 0.034)	0.061
High		0.059	1.208	0.341	3.546	(0.540, 1.875)	<0.001
Internship experience (reference: No)							
Yes		-0.001	-0.032	0.417	-0.078	(-0.850, 0.785)	0.938
Postgraduate entrance examination intention (reference: No)							
Yes		-0.006	-0.120	0.299	-0.403	(-0.706, 0.465)	0.687
University (reference: University A)							
University B		0.033	0.741	0.422	1.755	(-0.087, 1.569)	0.079
University C		-0.009	-0.198	0.436	-0.455	(-1.053, 0.656)	0.649
University D		0.048	1.107	0.436	2.540	(0.253, 1.960)	0.011

Note. The main-effects model is inserted between the mediator model and the interaction model. For the main-effects model, only the focal predictors are displayed; the same covariates and university fixed effects as in the interaction model were included. Overall model significance was evaluated using HC3 robust Wald F tests, and coefficient-level inference was based on HC3 heteroskedasticity-consistent standard errors. Grade, family economic status, academic pressure, and university affiliation were entered as categorical variables using dummy coding. University A was the reference category.

Supplementary Table S11. Simple slopes of intentional self-regulation predicting employment anxiety at different levels of perceived employability.

Perceived employability	*B*	HC3 *SE*	*t*	95% *CI*	*P*
Low, −1 SD	−0.150	0.012	−13.020	(−0.173, −0.128)	<0.001
Mean	−0.103	0.008	−12.440	(−0.119, −0.087)	<0.001
High, +1 SD	−0.055	0.012	−4.687	(−0.078, −0.032)	<0.001

Note. Simple slopes were estimated at the mean and at one standard deviation below and above the mean of perceived employability. All models included university fixed effects and were adjusted for age, sex, grade, residence, only-child status, family economic status, academic pressure, internship experience, and postgraduate entrance examination intention. HC3 robust standard errors were used. CI, confidence interval; SD, standard deviation.

Supplementary Table S12. Leave-one-university-out sensitivity analyses.

Excluded university	N	a path	b path	Direct c'	Indirect	Indirect 95% bootstrap CI	Interaction B	Interaction 95% *CI*	Interaction *P*
University A	2936	0.342	-0.099	-0.169	-0.034	(-0.042, -0.026)	0.005	(0.003, 0.007)	<0.001
University B	2823	0.364	-0.112	-0.168	-0.041	(-0.050, -0.032)	0.005	(0.003, 0.006)	<0.001
University C	2896	0.377	-0.097	-0.169	-0.037	(-0.047, -0.028)	0.004	(0.002, 0.006)	<0.001
University D	2916	0.379	-0.099	-0.164	-0.037	(-0.046, -0.029)	0.004	(0.003, 0.006)	<0.001

Note. The a path represents perceived school climate predicting intentional self-regulation; the b path represents intentional self-regulation predicting employment anxiety; direct c′ represents the direct association between perceived school climate and employment anxiety after including intentional self-regulation. Indirect effects and confidence intervals were estimated using 5,000 bootstrap resamples. The interaction is intentional self-regulation × perceived employability.
